# Attenuation and efficacy of human parainfluenza virus type 1 (HPIV1) vaccine candidates containing stabilized mutations in the P/C and L genes

**DOI:** 10.1186/1743-422X-4-67

**Published:** 2007-07-02

**Authors:** Emmalene J Bartlett, Adam Castaño, Sonja R Surman, Peter L Collins, Mario H Skiadopoulos, Brian R Murphy

**Affiliations:** 1 Laboratory of Infectious Diseases, Respiratory Viruses Section, National Institute of Allergy and Infectious Diseases (NIAID), National Institutes of Health (NIH), Department of Health and Human Services, Bethesda, MD, USA

## Abstract

**Background:**

Two recombinant, live attenuated human parainfluenza virus type 1 (rHPIV1) mutant viruses have been developed, using a reverse genetics system, for evaluation as potential intranasal vaccine candidates. These rHPIV1 vaccine candidates have two non-temperature sensitive (non-*ts*) attenuating (*att*) mutations primarily in the P/C gene, namely C^R84G^HN^T553A ^(two point mutations used together as a set) and C^Δ170 ^(a short deletion mutation), and two *ts att *mutations in the L gene, namely L^Y942A ^(a point mutation), and L^Δ1710–11 ^(a short deletion), the last of which has not been previously described. The latter three mutations were specifically designed for increased genetic and phenotypic stability. These mutations were evaluated on the HPIV1 backbone, both individually and in combination, for attenuation, immunogenicity, and protective efficacy in African green monkeys (AGMs).

**Results:**

The rHPIV1 mutant bearing the novel L^Δ1710–11 ^mutation was highly *ts *and attenuated in AGMs and was immunogenic and efficacious against HPIV1 wt challenge. The rHPIV1-C^R84G/Δ170^HN^T553A^L^Y942A ^and rHPIV1-C^R84G/Δ170^HN^T553A^L^Δ1710–11 ^vaccine candidates were highly *ts*, with shut-off temperatures of 38°C and 35°C, respectively, and were highly attenuated in AGMs. Immunization with rHPIV1-C^R84G/Δ170^HN^T553A^L^Y942A ^protected against HPIV1 wt challenge in both the upper and lower respiratory tracts. In contrast, rHPIV1-C^R84G/Δ170^HN^T553A^L^Δ1710–11 ^was not protective in AGMs due to over-attenuation, but it is expected to replicate more efficiently and be more immunogenic in the natural human host.

**Conclusion:**

The rHPIV1-C^R84G/Δ170^HN^T553A^L^Y942A ^and rHPIV1-C^R84G/Δ170^HN^T553A^L^Δ1710–11 ^vaccine candidates are clearly highly attenuated in AGMs and clinical trials are planned to address safety and immunogenicity in humans.

## Background

Human parainfluenza virus type 1 (HPIV1) is responsible for approximately 6% of pediatric hospitalizations due to respiratory tract disease with significant illness occurring predominantly in infants and young children [1]. Clinical manifestations range from mild disease, including rhinitis, pharyngitis, and otitis media, to more severe disease, including croup, bronchiolitis, and pneumonia [1-6]. Collectively, human parainfluenza virus serotypes 1, 2 and 3 (HPIV1, 2 and 3) are the second leading causative agents of pediatric hospitalizations due to respiratory disease following respiratory syncytial virus (RSV) [7,1]. However, a licensed vaccine is currently not available for the prevention of illness caused by any HPIV.

HPIV1 is an enveloped, non-segmented, single-stranded, negative-sense RNA virus belonging to the family *Paramyxoviridae*, genus *Respirovirus*, of which HPIV3 is also a member. The HPIV1 genome is 15,600 nucleotides in length and contains six genes in the order 3'-N-P/C-M-F-HN-L-5', which encode three nucleocapsid-associated proteins including the nucleocapsid protein (N), the phosphoprotein (P), and the large polymerase protein (L) and three envelope-associated proteins including the internal matrix protein (M) and the fusion (F) and hemagglutinin-neuraminidase (HN) transmembrane surface glycoproteins [8]. F and HN are the two viral neutralization antigens and are major viral protective antigens. The P/C gene of HPIV1 contains a second open reading frame (ORF) that encodes up to four accessory C proteins, C', C, Y1 and Y2, that initiate at four separate translational start codons in the C ORF and are carboxy co-terminal [1]. However, it is unclear whether the Y2 protein is actually expressed during HPIV1 infection [9]. The HPIV1 C proteins have recently been shown to act as antagonists of the innate immune response during virus infection by inhibiting type 1 interferon (IFN) production and signaling of IFN through its receptor [10].

Our laboratory is developing a live attenuated virus vaccine for HPIV1 for intranasal administration to infants and young children. The intranasal route of administration is needle-free and has the advantage of direct stimulation of local immunity as well as induction of a substantial systemic immune response [11]. Furthermore, compared to an inactivated vaccine, a live virus vaccine stimulates a broader spectrum of innate and adaptive immune responses [11]. The recent licensure of the trivalent live attenuated influenza virus vaccine (Flumist™) indicates that it is possible to achieve an acceptable balance between attenuation and immunogenicity with a live attenuated respiratory virus vaccine [12].

Reverse genetics provides a method for introducing attenuating mutations in desired combinations into wild type (wt) HPIV1 [13-16]. Temperature sensitive (*ts*) attenuating (*att*) and non-*ts att *mutations have been developed that, in combination, can enhance both the phenotypic and genetic stability of a HPIV1 vaccine candidate. The licensed cold-adapted influenza A viruses contain similar non-*ts *and *ts att *mutations [17,18]. In the case of HPIV1, non-*ts att *mutations have been introduced into the P/C gene that inactivate the anti-IFN activities of the C accessory proteins [10]. One of these mutations (C^Δ170^) is a deletion mutation that affects codon 170 of the HPIV1 C protein; deletion mutations are desirable because they are essentially free of same-site reversion and thus provide for enhanced genetic and phenotypic stability. The C^Δ170 ^mutation inhibited both the production of Type 1 IFN and the signaling of IFN through its receptor and specified an *att *phenotype in hamsters and African green monkeys (AGMs) [10,16]. A second non-*ts att *mutation involves a pair of amino acid substitutions, C^R84G ^and HN^T553A^, that attenuates HPIV1 for AGMs when they are present together but not individually. This attenuating pair of mutations was not further genetically stabilized, i.e., it is possible to revert to a wt phenotype with a single nucleotide substitution at either mutation. A substitution at amino acid position 942 of L, L^Y942A^, generated a *ts att *mutation that was engineered for increased genetic and phenotypic stability by the strategy of identifying a codon whose amino acid assignment yielded a *ts att *phenotype and which would require three nucleotide substitutions for reversion [13]. A virus bearing this stabilized mutation was attenuated in both AGMs and hamsters [15].

The present study consists of two parts. First, we developed an additional *ts att *mutation involving a small deletion in the HPIV1 L protein. This mutation was originally identified as a *ts att *point mutation in the bovine PIV3 (BPIV3) L protein (L^S1711I^) [19]. The corresponding site in the HPIV1 L protein was identified as position 1710 by sequence alignment, and this codon and its downstream neighbor (codon 1711) were deleted to yield the L^Δ1710–11 ^mutation. This gave us two genetically stabilized *ts att *mutations in L, the L^Δ1710–11 ^and the L^Y942A ^mutations. In the second part of the study, the two non-*ts att *mutations in C, namely the C^R84G^/HN^T553A ^set and the C^Δ170 ^mutation, were combined with each other and with either the L^Δ1710–11 ^mutation or the L^Y942A ^mutation to develop two live intranasal HPIV1 vaccine candidates. Each of these vaccine candidates contained at least one genetically stabilized *ts *and non-*ts att *mutation. These viruses were evaluated for their in vitro attenuation phenotype and for replication, efficacy and immunogenicity in AGMs.

## Results

### Construction and recovery of mutant rHPIV1 viruses

Point and deletion mutations in the P/C, HN and L genes that attenuate HPIV1 for replication in the respiratory tract of hamsters or AGMs are indicated in Table 1[13-16]. The C^R84G ^mutation is a single nucleotide substitution mutation that affects both the P and C proteins and that results in amino acid substitutions of R84 to G in C, and E87 to G in P (Table 1) [15]. The C^R84G ^mutation is attenuating in the upper respiratory tract (URT) of AGMs, but only in the presence of the HN^T553A ^point mutation indicated in Table 1[15]. The C^R84G ^and HN^T553A ^mutations are each based on single nucleotide substitutions (Table 1), and thus the *att *phenotype would be lost by reversion at either position. The C^Δ170 ^deletion mutation in HPIV1 involves a six-nucleotide deletion, a length that was chosen to comply with the "rule of six" [20]. This deletion results in a loss of two amino acids and substitution of a third at codon positions 168–170 in C (RDF to S), and a deletion of amino acids GF in P at codon positions 172–173 (Table 1) [16]. The changes in the C protein also would be present in the nested C', Y1, and Y2 proteins (not shown) [16]. The Y942A mutation in L has three nucleotide changes in codon 942 and specifies a genetically and phenotypically stabilized *ts att *phenotype [13].

**Table 1:** Summary of the mutations introduced into the rHPIV1 genome^a^.

**Gene**	**Mutation**^b^	**ORF**	**nt changes wt → mutant **^c^	**Type of mutation**	**Codon position**	**Amino acid change**	**# nt changes for reversion to wt**
P/C	R84G	C	**A**GA → **G**GA	point	84	R → G	1
		P	G**A**G → G**G**G	point	87	E → G	1
	Δ170^d^	C	AG**G GAT TT**C → AGC	deletion	168–170	RDF → S (D deletion; 3 nt deletions in the flanking R-F codons results in a S substitution)	6 (insertions)^d^
		P	**GGA TTT**→ deletion	deletion	172–173	GF deletion	6 (insertions)
HN	T553A	HN	**A**CC → **G**CC	point	553	T → A	1
L	Y942A^e^	L	**TAT**→ **GCG**	point	942	Y → A	3^e^
	Δ1710–11^d^	L	**GCT GAG**→ deletion	deletion	1710–11	AE deletion	6 (insertions)^d^

^a ^HPIV1 strain Washington/1964, GenBank accession no. NC_003461.

^b ^The nomenclature used to describe each mutation indicates the wt amino acid, the codon position and the new amino acid, or the position of the deletion (Δ), with respect to the C, HN or L protein.

^c ^The nucleotides (nt) affected by substitution or deletion are shown underlined and in bold type.

^d ^Designed for increased genetic stability by use of a deletion. Deletions involved six nt to conform to the rule of six [20].

^e ^Designed for increased genetic stability by the use of a codon that differs by three nucleotides from codons yielding a wild type assignment.

In the present study, the L^Δ1710–11 ^deletion mutation in HPIV1 was created at a site that corresponds by sequence alignment to a *ts att *point mutation originally identified in BPIV3 [19]. Importation of this BPIV3 point mutation has previously been shown to attenuate HPIV2 [21]. Here, the L^Δ1710–11 ^mutation contains a six-nucleotide deletion that results in a deletion of amino acids AE at codon positions 1710–11 of the L gene of HPIV1 (Table 1).

The mutations in Table 1 were introduced into the HPIV1 antigenomic cDNA individually or in combinations to yield the panel of rHPIV1 viruses listed in Table 2. These viruses were recovered following transfection of cDNAs into HEp-2, BHK-T7 or Vero cells and biologically cloned in LLC-MK2 cells, and each was sequenced in its entirety to confirm the presence of the engineered mutation(s) and the absence of adventitious mutations. Unexpectedly, we were unable to isolate rHPIV1 containing the L^Δ1710–11 ^mutation by itself and without adventitious mutations despite four attempts to do this using multiple replicates each time. However, we were able to recover virus bearing L^Δ1710–11 ^in the presence of C^R84G ^without adventitious mutations. Thus, our analysis of the phenotype of the L^Δ1710–11 ^mutation was performed in the presence of the C^R84G ^mutation, which is neither *ts *nor *att *[15].

**Table 2:** Level of temperature sensitivity of replication of rHPIV1 mutants in vitro.

			**Mean reduction (log_10_) in virus titer ± S.E. at the indicated temperature compared to 32°C **^c^						
				
**Virus **^a^		**Virus titer ± S.E. at 32°C **^b^	**35°C**	**36°C**	**37°C**	**38°C**	**39°C**	**40°C**	**Shut-off (°C) **^d^
1	HPIV1 wt	7.7 ± 0.1	0.1 ± 0.1	0.1 ± 0.1	0.2 ± 0.1	0.7 ± 0.1	1.3 ± 0.1	3.0 ± 0.3	-
2^e^	rHPIV1-C^R84G^	9.2 ± 0.4	0.4 ± 0.2	0.4 ± 0.6	0.8 ± 0.5	0.3 ± 0.4	1.8 ± 0.6	4.5 ± 0.9	-
3^e^	rHPIV1-C^R84G^HN^T553A^	7.8 ± 0.1	-0.3 ± 0.2	-0.3 ± 0.2	-0.2 ± 0.2	0.1 ± 0.2	0.7 ± 0.2	2.5 ± 0.6	-
4^e^	rHPIV1-C^Δ170^	7.9 ± 0.3	0.2 ± 0.2	0.7 ± 0.8	0.5 ± 0.2	1.0 ± 0.3	2.6 ± 0.7	4.5 ± 1.0	-
5	rHPIV1-L^Y942A^	8.0 ± 0.1	0.2 ± 0.3	1.2 ± 0.3	**2.6 ± 1.1**^c,d^	**6.4 ± 0.4**	≥**6.8 **^f^	≥**6.8**	37°C
6^e^	rHPIV1-C^R84G^HN^T553A^L^Y942A^	7.4 ± 0.2	0.4 ± 0.4	0.5 ± 0.4	**2.3 ± 0.4**	**4.0 ± 0.6**	**6.0 ± 0.4**	≥**6.4**	37°C
7	rHPIV1-C^R84G^L^Δ1710–11^	7.5 ± 0.7	0.8 ± 0.7	**3.0 ± 0.6**	**4.8 ± 0.2**	≥**6.3**	≥**6.3**	≥**6.3**	36°C
8	rHPIV1-C^R84G/Δ170^HN^T553A^L^Y942A^	6.3 ± 0.1	0.3 ± 0.2	0.9 ± 0.6	2.0 ± 0.3	**4.9 ± 0.2**	≥**5.1**	≥**5.1**	38°C
9	rHPIV1-C^R84G/Δ170^HN^T553A^L^Δ1710–11^	6.4 ± 0.3	**2.6 ± 0.6**	**4.0 ± 0.4**	≥**5.2**	≥**5.2**	≥**5.2**	≥**5.2**	35°C

^a ^Data are the mean of three to sixteen experiments.

^b ^Viruses were titrated on LLC-MK2 cells at either permissive (32°C) or potentially restrictive (35°C – 40°C) temperatures for 7 days and virus titers are expressed as the mean ± standard error (S.E.). The limit of detection was 1.2 log_10 _TCID_50_/ml.

^c ^Values in bold indicate restricted replication, where the mean log_10 _reduction in virus titer at the indicated temperature vs 32°C was 2.0 log_10 _or greater than the difference in titer of HPIV1 wt at the same temperature vs 32°C. A virus is designated *ts *if restricted replication at 35°C–40°C is observed.

^d ^Underlined values indicate viral shut-off temperature, the lowest temperature at which restricted replication is observed.

^e ^These data have been previously published [13] [15] [16] and are included here for the purposes of comparison.

^f ^The symbol "≥" indicates that virus titers were at the limit of detection and therefore the reduction in virus titer versus 32°C is greater than or equal to the indicated value. There is no S.E. value for viruses at the limit of detection.

### Characterization of rHPIV1s containing single att mutations

We first sought to characterize the rHPIV1 mutants bearing the four single *att *mutations (the C^R84G^HN^T553A ^set, C^Δ170^, L^Y942A^, and L^Δ1710–11^) to define the contributions of the individual mutations to the phenotypes of the rHPIV1 mutants (Groups 3, 4, 5, 7 in Tables 2 and 3). We previously generated and evaluated the rHPIV1-C^R84G^HN^T553A ^and rHPIV1-C^Δ170 ^viruses (each containing a single non-*ts att *mutation) in vitro and in vivo [13,15,16]. These previously evaluated single-mutation viruses were included here for the purpose of comparison with viruses containing the other individual mutations as well as combinations of mutations. An rHPIV1 mutant, rHPIV1-L^Y942A^, bearing the Y942A mutation in L was generated for the present study. We had previously generated and characterized a virus, rHPIV1-C^R84G^HN^T553A^L^Y942A^, containing the L^Y942A ^mutation in combination with the C^R84G^HN^T553A ^pair of mutations [13]. The newly generated rHPIV1-L^Y942A ^virus would permit evaluation of its specific contribution to the level of temperature sensitivity in vitro and attenuation in vivo. The rHPIV1 mutant bearing the individual *att *mutation L^Δ1710–11 ^(rHPIV1-C^R84G^L^Δ1710–11^) also contained the C^R84G ^mutation, although this latter mutation is phenotypically silent on its own, as already noted.

**Table 3:** Level of replication of HPIV1 vaccine candidates in the upper and lower respiratory tract of African green monkeys.

				**Mean peak virus titer (log_10 _TCID_50_/ml)**^c^		**Mean sum of the daily virus titers (log_10 _TCID_50_/ml)**^d^		***att***^e^	
						
**Virus**^a^		**Shut-off temperature **^b^	**No. of animals**	**NP swab**^f^	**TL **^g^	**NP swab**^f^	**TL **^g^	**URT**	**LRT**
1	HPIV1 wt	-	14	4.2 ± 0.2	3.9 ± 0.3	26.4 ± 1.5	12.2 ± 1.6	-	-
2^h^	rHPIV1-C^R84G^	-	4	3.6 ± 0.4	4.0 ± 0.5	21.0 ± 1.7	11.7 ± 2.5	No	No
3^h^	rHPIV1-C^R84G^HN^T553A^	-	12	2.1 ± 0.2^i^	4.8 ± 0.3	10.5 ± 0.9	14.3 ± 1.1	Yes	No
4^h^	rHPIV1-C^Δ170^	-	6	3.4 ± 0.5	2.3 ± 0.5	14.8 ± 1.9	5.1 ± 0.8	Yes	Yes
5	rHPIV1-L^Y942A^	37°C	4	2.3 ± 0.1	2.3 ± 0.2	16.9 ± 0.7	8.4 ± 1.2	Yes	Yes
6^h^	rHPIV1-C^R84G^HN^T553A^L^Y942A^	37°C	8	2.4 ± 0.2	2.1 ± 0.3	12.9 ± 1.0	5.1 ± 0.6	Yes	Yes
7	rHPIV1-C^R84G^L^Δ1710–11^	36°C	4	1.5 ± 0.4	0.9 ± 0.2	8.6 ± 1.8	3.2 ± 0.6	Yes	Yes
8	rHPIV1-C^R84G/Δ170^HN^T553A^L^Y942A^	38°C	4	1.2 ± 0.3	0.6 ± 0.1	5.9 ± 0.5	2.6 ± 0.1	Yes	Yes
9	rHPIV1-C^R84G/Δ170^HN^T553A^L^Δ1710–11^	35°C	4	0.9 ± 0.3	≤0.5 ± 0.0	6.3 ± 0.5	≤2.5 ± 0.0	Yes	Yes

^a ^Monkeys were inoculated i.n. and i.t. with 10^6 ^TCID_50 _of the indicated virus in a 1 ml inoculum at each site. Data are representative of one to five experiments.

^b ^Shut-off temperature is defined in footnote d, Table 2.

^c ^Virus titrations were performed on LLC-MK2 cells at 32°C and expressed as the mean ± S.E of the individual peak virus titers for the animals in each group irrespective of day. The limit of detection was 0.5 log_10 _TCID_50_/ml.

^d ^Mean sum of the daily virus titers: the sum of the titers for all of the days of sampling was determined for each animal individually, and the mean was calculated for each group. On days when virus was not detected, a value of was 0.5 log_10 _TCID_50_/ml was assigned for the purpose of calculation. The mean sum of the lower limit of detection was 5.0 log_10 _TCID_50_/ml for NP swabs and 2.5 log_10 _TCID_50_/ml for TL samples.

^e ^Virus is designated *att *in the URT or LRT based on a significant reduction in either mean peak titer or mean sum of daily titers compared to the HPIV1 wt group (see footnote h).

^f ^Nasopharyngeal (NP) swab samples were collected on days 1–10 post-infection.

^g ^Tracheal lavage (TL) samples were collected on days 2, 4, 6, 8, and 10 post-infection.

^h ^These data have been previously published [13] [15] [16] and are included here for the purposes of comparison.

^i ^Underlined values indicate a statistically significant reduction compared to corresponding HPIV1 wt titer, *P *< 0.05 (Student-Newman-Keuls multiple comparison test).

**Figure 1:**
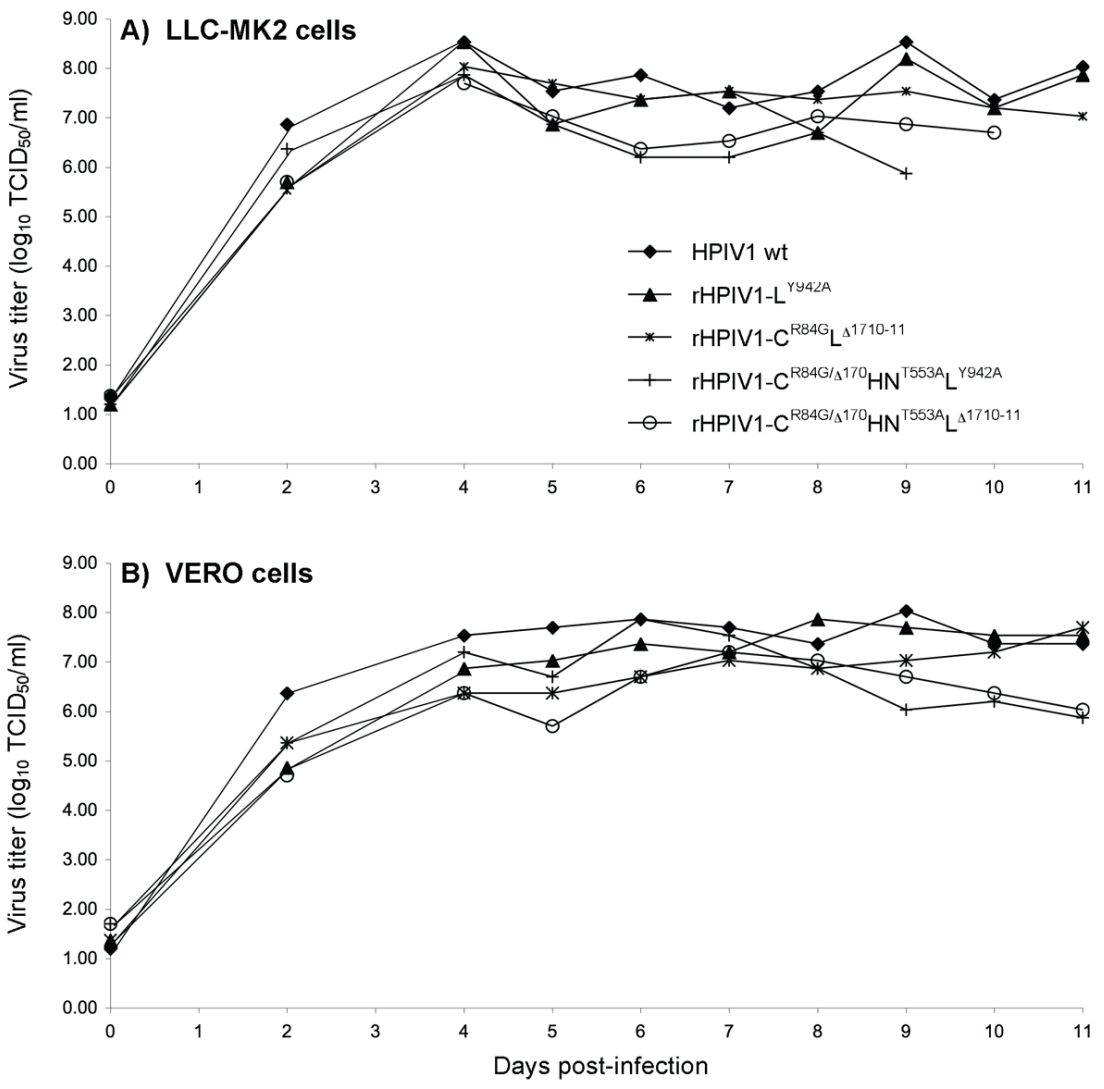
**Comparison of the replication of HPIV1 wt and rHPIV1 mutant viruses containing the indicated mutations in the P/C, HN and L genes in a multiple cycle growth curve**. Monolayer cultures of LLC-MK2 cells and Vero cells were infected at a multiplicity of infection of 0.01 TCID_50_/cell and incubated at 32°C. The medium was removed on days 0 (residual inoculum), 2 and 4–11 post-infection, frozen for later determination of virus titers, and replaced by fresh medium containing trypsin. The virus titers shown are the means of 3 replicate cultures.

The level of temperature sensitivity of replication of the four viruses with single *att *mutations was first studied (Table 2, groups 3, 4, 5, and 7) and compared to that of rHPIV1 wt and rHPIV1-C^R84G^. Viruses containing only P/C gene mutations with or without the HN mutation were non-*ts*, whereas each of the L gene mutations specified a *ts *phenotype in vitro. The single L^Y942A ^mutation specified a shut-off temperature of 37°C, a level of temperature sensitivity that was equivalent to that previously observed for rHPIV1-C^R84G^HN^T553A^L^Y942A ^(Table 2, compare Groups 5 and 6). These data indicate that the L^Y942A ^mutation is responsible for the observed *ts *phenotype of rHPIV1-C^R84G^HN^T553A^L^Y942A ^(Table 2). The L^Δ1710–11 ^mutation specified an even stronger *ts *phenotype than the L^Y942A ^mutation (Table 2). The L^Δ1710–11 ^mutation clearly contributes significantly to the *ts *property of rHPIV1-C^R84G^L^Δ1710–11 ^since rHPIV1-C^R84G ^was confirmed to be non-*ts *(Table 2, compare Groups 2 and 7). Therefore, both L^Y942A ^and L^Δ1710–11 ^are *ts *mutations in HPIV1. In a multiple cycle growth curve, the two newly generated rHPIV1 mutants with single *att *mutations, rHPIV1-L^Y942A ^and rHPIV1-C^R84G^L^Δ1710–11^, reached a titer equivalent to that of rHPIV1 wt in both LLC-MK2 and Vero cells (Figure 1). Thus, these individual mutations do not significantly restrict replication in vitro at the permissive temperature of 32°C and therefore could be useful mutations in vaccine candidates.

The level of replication of rHPIV1-L^Y942A ^and rHPIV1-C^R84G^L^Δ1710–11 ^in AGMs was next evaluated and compared to that of rHPIV1 wt and the other two single *att *mutants (Table 3, Groups 1, 3, 4, 5, 7). A rHPIV1 mutant was considered attenuated if it exhibited a significant (*P *< 0.05) reduction in replication in either the mean peak virus titer or the mean sum of the daily virus titers (a measure of the total amount of virus shed over the duration of the infection) in either the nasopharyngeal (NP) swab (representative of the upper respiratory tract, URT) or tracheal lavage (TL) samples (representative of the lower respiratory tract, LRT) compared to the HPIV1 wt group. We have previously demonstrated that rHPIV1-C^R84G ^replicates to levels equivalent to HPIV1 wt in AGMs, whereas rHPIV1-C^R84G^HN^T553A ^and rHPIV1-C^R84G^HN^T553A^L^Y942A ^were attenuated in AGMs [15,16]. Here, both rHPIV1-L^Y942A ^and rHPIV1-C^R84G^L^Δ1710–11 ^were significantly attenuated in the URT and LRT of AGMs in comparison to HPIV1 wt. The levels of attenuation of rHPIV1-L^Y942A ^and rHPIV1-C^R84G^HN^T553A^L^Y942A ^were comparable, indicating that the L^Y942A ^mutation is an attenuating mutation by itself and that the attenuation specified by the L^Y942A ^mutation is not additive to that specified by the C^R84G^HN^T553A ^*att *mutation. The rHPIV1-C^R84G^L^Δ1710–11 ^mutant also was significantly attenuated in AGMs, reducing virus titer in comparison to HPIV1 wt by 2.7 and 3.0 log_10 _50%-tissue-culture-infectious-doses (TCID_50_)/ml in the URT and LRT, respectively (Table 3). Since rHPIV1-C^R84G ^was confirmed not to be attenuated in AGMs (Table 3, Group 2) [16], this suggests that the L^Δ1710–11 ^mutation contributes significantly to the observed attenuation phenotype.

The immunogenicity and protective efficacy resulting from immunization with rHPIV1s containing single *att *mutations were evaluated in AGMs by measuring post-immunization HPIV1 hemagglutination inhibiting (HAI) serum antibody titers and by challenging immunized and control animals with HPIV1 wt 28 days following immunization and determining challenge virus titers in the URT and LRT (Table 4). AGMs immunized with rHPIV1s containing single *att *mutations (Groups 3, 4, 5, and 7) developed post-immunization HAI serum antibodies and manifested resistance to replication of the challenge virus. The rHPIV1-C^R84G^L^Δ1710–11 ^mutant, which showed a strong level of attenuation following immunization of AGMs, was protective only at a low level in the URT.

**Table 4:** Immunogenicity and protective efficacy of rHPIV1 vaccine candidates in AGMs.

				**Mean peak challenge virus titer (log_10_TCID_50_/ml) **^c^		**Mean sum of the daily challenge virus titers (log_10_TCID_50_/ml)**^d^		**Post-challenge serum HAI titer**^b^
						
**Virus **^a^		**No. animals**	**Pre-challenge serum HAI titer**^b^	**NP swab**	**TL**	**NP swab**	**TL**	
1	HPIV1 wt	12	6.7 ± 0.6 (12/12)	0.8 ± 0.2^f^	0.7 ± 0.1	2.3 ± 0.2	2.4 ± 0.2	6.6 ± 0.5
2^e^	rHPIV1-C^R84G^	4	3.8 ± 0.9 (3/4)	≤0.5 ± 0.0	≤0.5 ± 0.0	≤2.0 ± 0.0	≤2.0 ± 0.0	4.4 ± 1.2
3^e^	rHPIV1-C^R84G^HN^T553A^	12	6.0 ± 0.6 (11/12)	0.6 ± 0.1	0.6 ± 0.1	2.1 ± 0.1	2.1 ± 0.1	7.9 ± 0.4
4^e^	rHPIV1-C^Δ170^	6	5.5 ± 0.4 (6/6)	≤0.5 ± 0.0	≤0.5 ± 0.0	≤2.0 ± 0.0	≤2.0 ± 0.0	6.5 ± 0.4
5	rHPIV1-L^Y942A^	4	6.3 ± 1.2 (4/4)	1.1 ± 0.2	1.2 ± 0.2	2.7 ± 0.3	2.8 ± 0.3	8.9 ± 1.1
6^e^	rHPIV1-C^R84G^HN^T553A^L^Y942A^	8	2.0 ± 0.0 (3/8)	0.8 ± 0.2	0.8 ± 0.2	2.6 ± 0.3	2.4 ± 0.3	3.3 ± 0.7
7	rHPIV1-C^R84G^L^Δ1710–11^	4	6.1 ± 1.8 (3/4)	3.4 ± 0.6	3.0 ± 0.6	8.4 ± 2.0	8.3 ± 1.3	6.9 ± 1.5
8	rHPIV1-C^R84G/Δ170^HN^T553A^L^Y942A^	4	≤1.0 ± 0.0 (0/4)	2.2 ± 0.2	1.8 ± 0.5	5.1 ± 0.3	4.3 ± 1.3	5.5 ± 1.6
9	rHPIV1-C^R84G/Δ170^HN^T553A^L^Δ1710–11^	4	≤1.0 ± 0.0 (0/4)	4.5 ± 0.9	3.4 ± 0.4	11.8 ± 2.5	8.1 ± 1.3	7.5 ± 1.4
10	Non-immune	7	≤1.0 ± 0.0 (0/4)	5.0 ± 0.6	3.9 ± 0.5	14.8 ± 1.2	11.0 ± 2.5	6.0 ± 1.3

^a ^Monkeys were immunized i.n. and i.t. with 10^6 ^TCID_50 _of the indicated virus in a 1 ml inoculum at each site and were challenged on day 28 post-infection with HPIV1 wt.

^b ^HAI titers to HPIV1 were determined by HAI assay of sera collected at day 28 (pre-challenge) and day 56 (post-challenge) in separate assays. Titers are expressed as mean reciprocal log_2_± S.E.; the limit of detection was 1.0 ± 0.0. The number of animals with a 4-fold or greater increase in pre-challenge antibody titers is shown in brackets for each group.

^c ^Mean ± S.E of the individual peak virus titers for the animals in each group irrespective of day. Virus titrations were performed on LLC-MK2 cells at 32°C. The limit of detection was 0.5 log_10 _TCID_50_/ml. NP and TL samples were collected on days 2, 4, 6 and 8 post-challenge.

^d ^Mean sum of the daily virus titers: the sum of the titers for all of the days of sampling was determined for each animal individually, and the mean was calculated for each group. On days when no virus was detected, a value of was 0.5 log_10 _TCID_50_/ml was assigned for the purpose of calculation. The mean sum of the lower limit of detection was 2.0 log_10 _TCID_50_/ml for NP swabs and TL samples.

^e ^These data have been previously published [13] [15] [16] and are included here for the purposes of comparison.

^f ^Underlined values indicate statistically significant reductions in mean peaks or sum of daily virus titers for HPIV1 wt titer compared to the corresponding non-immune group, *P *< 0.05 (Student-Newman-Keuls multiple comparison test).

### Combination of three single att mutations into rHPIV1 to generate two live attenuated HPIV1 vaccine candidates

Having identified the in vitro and in vivo properties of the four single *att *mutations, we used this information to generate two live attenuated HPIV1 vaccine candidates containing both non-*ts *and *ts *attenuating mutations. These vaccine candidates were designed to incorporate a backbone containing one stabilized non-*ts *attenuating mutation, C^Δ170^, as well as the C^R84G^HN^T553A ^*att *mutation. The addition of this second mutation (the C^R84G^HN^T553A ^*att *mutation) would be expected to increase the overall stability of the virus by increasing the total number of attenuating mutations present in the vaccine candidate. To generate the two live attenuated HPIV1 vaccine candidates, either the stabilized *ts att *L^Y942A ^mutation or the L^Δ1710–11 ^deletion mutation was added to the rHPIV1-C^R84G/Δ170^HN^T553A ^backbone. We then evaluated the resulting combination mutants, rHPIV1-C^R84G/Δ170^HN^T553A^L^Y942A ^and rHPIV1-C^R84G/Δ170^HN^T553A^L^Δ1710–11^, as potential vaccine candidates.

These two viruses were first evaluated for their level of temperature sensitivity of replication in vitro (Table 2). The level of temperature sensitivity of rHPIV1-C^R84G/Δ170^HN^T553A^L^Y942A ^and rHPIV1-C^R84G/Δ170^HN^T553A^L^Δ1710–11 ^(Groups 8 and 9 in Table 2) was equivalent to that of the corresponding L gene single-mutation viruses from which they were derived (namely rHPIV1-L^Y942A ^and rHPIV1-C^R84G^L^Δ1710–1^, Groups 5 and 7 in Table 2). This indicates that combining the non-*ts *and *ts *mutations in rHPIV1-C^R84G/Δ170^HN^T553A^L^Y942A ^and rHPIV1-C^R84G/Δ170^HN^T553A^L^Δ1710–11 ^did not significantly alter their overall level of temperature sensitivity of replication in vitro. A multiple cycle growth curve at 32°C demonstrated that each virus achieved titers in Vero cells that will allow efficient manufacture. Specifically, the rHPIV1-C^R84G/Δ170^HN^T553A^L^Y942A ^and rHPIV1-C^R84G/Δ170^HN^T553A^L^Δ1710–11 ^vaccine candidates reached peak titers of 7.9 and 7.2 log_10 _TCID_50_/ml, respectively, in Vero cells (Figure 1).

**Figure 2:**
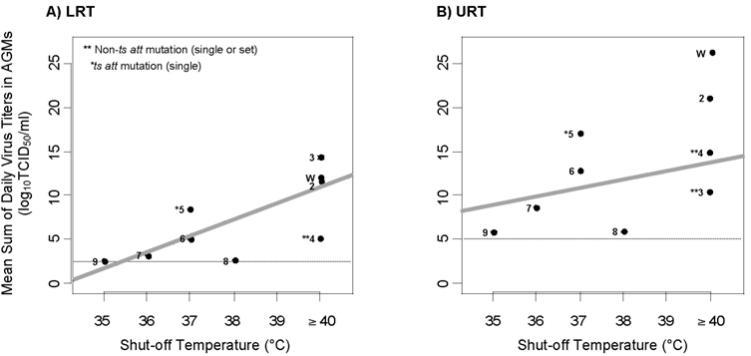
**Representation of the association between the in vitro shut-off temperature and the attenuation phenotype in AGMs for HPIV1 wt (W) and rHPIV1 mutant viruses**. For each virus (number designations correspond to the virus group numbers assigned in tables 2-4), the shut-off temperature (°C), as determined by an in vitro temperature sensitivity assay (Table 2), was plotted against the mean sum of daily virus titers (log_10 _TCID_50_/ml; Table 3) in the URT (A) and LRT (B) of AGMs. rHPIV1 wt and non-*ts *rHPIV1 mutants were assigned a shut-off temperature of 40°C for the purposes of this schematic. The limit of detection for the mean sum of daily virus titers is shown by a dashed line and viruses containing a single or set of non-*ts *attenuating mutation (**) or a single *ts *attenuating mutation (*) are highlighted, as shown. A linear trend line fit using the individual daily data is shown (solid line). The Spearman rank-correlation coefficient was determined to be 0.47 for the URT and 0.67 for the LRT, indicating a moderate positive correlation between shut-off temperature and mean daily sum of virus titer in the URT and a stronger association for the LRT.

The level of replication of rHPIV1-C^R84G/Δ170^HN^T553A^L^Y942A ^and rHPIV1-C^R84G/Δ170^HN^T553A^L^Δ1710–11 ^in AGMs were next evaluated and compared to that of rHPIV1 wt and the other two single *att *mutants (Table 3, Groups 1, 3, 4, 5, 7, 8, and 9). The rHPIV1-C^R84G/Δ170^HN^T553A^L^Y942A ^virus was strongly attenuated compared to rHPIV1 mutants bearing the corresponding single *att *mutations only in C/P, C/P/HN or L. The mean peak titer of rHPIV1-C^R84G/Δ170^HN^T553A^L^Y942A ^in the URT and LRT was reduced by 3.0 and 3.3 log_10 _TCID_50_/ml, respectively, in comparison to HPIV1 wt (Table 3). Similarly, the addition of the HN^T553A ^and C^Δ170 ^mutations to rHPIV1-C^R84G^L^Δ1710–11 ^to generate the rHPIV1-C^R84G/Δ170^HN^T553A^L^Δ1710–11 ^further attenuated the virus in AGMs, restricting virus replication in comparison to HPIV1 wt by 3.1 and 3.4 log_10 _TCID_50_/ml in the URT and LRT, respectively (Table 3). Therefore these two HPIV1 vaccine candidates demonstrate strong attenuation phenotypes in vivo. Considering the 9 viruses in Table 3 together, a relationship was found to exist between level of temperature sensitivity of replication in vitro and the attenuation manifested in vivo, i.e., the lower the shut off temperature, the higher the level of in vivo attenuation (Figure 2). Evaluation of these data using the Spearman rank test gives correlation coefficients of 0.47 and 0.67 for the URT and LRT, respectively, based on the mean daily sum of virus titers for individual AGMs. This indicates a moderate positive correlation with a stronger association between the level of temperature sensitivity and virus replication in the LRT. However, as might be expected, viruses bearing only the non-*ts *attenuating P/C gene mutations, including the C^Δ170 ^and the C^R84G^HN^T553A ^set of mutations, did not follow this pattern (Figure 2), and we would expect a higher correlation coefficient if these non-*ts *viruses were not included in the analysis.

**Figure 3:**
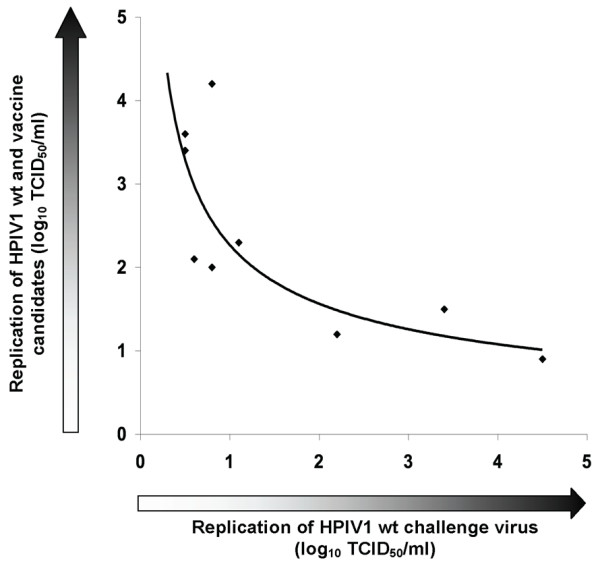
**Representation of the relationship between the level of replication of HPIV1 wt and rHPIV1 mutants in AGMs and the subsequent level of replication of HPIV1 wt challenge virus in the immunized animals**. The mean peak virus titer (log_10 _TCID_50_/ml) in the URT following immunization (y-axis) was plotted for viruses 1–9 (Table 3) against the mean peak challenge virus titers (log_10 _TCID_50_/ml; x-axis) in the same groups (Table 4). A curve of best fit has been inserted (solid line) to demonstrate the association between these two data sets.

The levels of immunogenicity and protective efficacy against HPIV1 wt challenge following immunization with rHPIV1-C^R84G/Δ170^HN^T553A^L^Y942A ^and rHPIV1-C^R84G/Δ170^HN^T553A^L^Δ1710–11 ^were also determined (Groups 8 and 9 in Table 4). The two vaccine candidates failed to induce detectable HAI antibodies. However, immunization with the rHPIV1-C^R84G/Δ170^HN^T553A^L^Y942A ^was protective against HPIV1 wt challenge in both the URT and LRT (Table 4). In contrast, immunization with rHPIV1-C^R84G/Δ170^HN^T553A^L^Δ1710–11 ^did not offer significant protection against HPIV1 wt challenge in the AGMs (Table 4), i.e., it appeared overattenuated in this animal model. A relationship was found between the level of replication of the immunizing virus and its ability to induce resistance to replication of the challenge virus (Tables 3 and 4), and this is graphically displayed in Figure 3.

## Discussion

The advent of a reverse genetics system for the generation of infectious paramyxoviruses from full-length cDNA plasmids has greatly facilitated the development of live attenuated HPIV1 vaccine candidates [13-16]. The reverse genetics system for HPIV1 has allowed site-directed manipulation of the viral genome via cDNA intermediates, permitting the introduction of attenuating mutations in desired combinations into vaccine candidates. It has also been possible to genetically modify some of the attenuating mutations to optimize genetic and phenotypic stability of viruses bearing the mutations, both by the use of gene deletions and by using codons chosen for a low probability of reversion. This process enables us to optimize the safety profile of the live attenuated HPIV1 vaccine candidates before these viruses are tested in humans.

We are focusing our efforts on the development of live attenuated rHPIV1 vaccines since they have a number of advantages over inactivated or subunit vaccines, including the ability to: (i) induce the full spectrum of protective immune responses including serum and local antibodies as well as CD4+ and CD8+ T cells [11]; (ii) infect and replicate in the presence of maternal antibody permitting immunization of young infants [22,23]; (iii) cause an acute, self-limited infection that is readily eliminated from the respiratory tract; and (iv) replicate to high titers in cell substrates acceptable for products for human use, including qualified Vero cells, making manufacture of these vaccines commercially feasible. In the present study, two new rHPIV1 viruses containing single *att *mutations in L, L^Δ1710–11 ^and L^Y942A^, were generated and characterized, and these *ts att *mutations were used in combination with previously described non-*ts att *mutations in the P/C gene and HN gene to generate two new live attenuated HPIV1 vaccine candidates.

A major result of the present study was the creation of the L^Δ1710–11 ^mutation that was found to specify a strong *ts att *phenotype. The L^Δ1710–11 ^mutation was originally identified as an attenuating point mutation, L^T1711I^, in BPIV3 [19]. It was evaluated as a deletion mutation in the present study since a deletion mutation offers a higher level of genetic stability than a point mutation, a property that is desirable for mutations in a vaccine candidate. Indeed, since this deletion occurs in an ORF (in which the triplet nature of the codons must be maintained) and in a virus that conforms to the rule of six (in which the hexamer organization must be maintained), same-site reversion would require the precise restoration of six nucleotides. We unfortunately were not able isolate a rHPIV1 mutant with only the L^Δ1710–11 ^mutation since each rHPIV1-L^Δ1710–11 ^mutant that was isolated also possessed one or more adventitious mutations. The L^Δ1710–11 ^mutation could only be recovered free of adventitious mutations when it was in combination with the C^R84G ^mutation, and thus had to be studied in that context. We acknowledge that it is possible that the phenotypes that we observed for the rHPIV1-C^R84G^L^Δ1710–11 ^are the result of an interaction between the C^R84G ^and L^Δ1710–11 ^mutations. However, we believe that this possibility is unlikely since the C^R84G ^mutation does not contribute to the *ts *or *att *phenotype of HPIV1 as an independent mutation. Furthermore, the high level of temperature sensitivity and attenuation of rHPIV1-C^R84G^L^Δ1710–11 ^versus that of rHPIV1-C^R84G ^suggests a major independent role of the L^Δ1710–11 ^mutation in these two phenotypes. rHPIV1-C^R84G^L^Δ1710–11 ^manifested a shut-off temperature of 37°C in vitro and was restricted in replication in the URT and LRT of AGMs by 2.5 log_10 _or 3.0 log_10_, respectively. Therefore, we suggest that the L^Δ1710–11 ^deletion mutation specifies a *ts att *phenotype for HPIV1, and, as such, it is a suitable mutation to include in a HPIV1 vaccine candidate.

The L^Y942A ^mutation was identified previously as an attenuating mutation for introduction into potential HPIV1 vaccine candidates and was stabilized by codon optimization studies [13]. These studies demonstrated that only three amino acids were shown to specify a wild type phenotype at this codon position (the wild type tyrosine, cysteine and phenylalanine) all of which would require three nucleotide changes to convert the GCG alanine to a codon specifying the wild type phenotype codon in the vaccine virus [13]. In addition, the L^Y942A ^mutation was shown to be highly stable under selective pressure during passage at permissive and restrictive temperatures [13]. Previous studies have evaluated the L^Y942A ^mutation only in the presence of the C^R84G^HN^T553A ^set of mutations that attenuates HPIV1 for AGMs [13,15]. To determine the specific contribution of the L^Y942A ^mutation to the *ts *and *att *phenotypes associated with the rHPIV1-C^R84G^HN^T553A^L^Y942A ^virus, a rHPIV1 containing only the L^Y942A ^mutation was generated and was found to be as attenuated as rHPIV1-C^R84G^HN^T553A^L^Y942A ^for AGMs. This indicated that the L^Y942A ^mutation independently attenuated HPIV1 for AGMs and can be used in the absence of the C^R84G^HN^T553A ^mutation to attenuate HPIV1 for AGMs. The attenuation specified by the C^R84G^HN^T553A ^mutation was not additive with that of L^Y942A^. This actually is a desirable property, since it permits the inclusion of a greater number of mutations while avoiding over-attenuation, and these additional mutations would become unmasked in the case of the loss of one or more other mutations and would thus maintain the *att *phenotype. Thus, L^Y942A ^is a stable mutation that specifies a *ts att *phenotype for HPIV1 and is suitable for introducing into a HPIV1 vaccine candidate as an independent attenuating mutation.

The L^Y942A ^and L^Δ1710–11 ^*ts att *mutations were used in conjunction with two of the non-*ts att *mutations, the C^R84G^HN^T553A ^and C^Δ170 ^mutations [16], to develop two live attenuated vaccine candidates for HPIV1, namely, rHPIV1-C^R84G/Δ170^HN^T553A^L^Y942A ^and rHPIV1-C^R84G/Δ170^HN^T553A^L^Δ1710–11^. These vaccine candidates thus each contain three independent attenuating mutations (two non-*ts att *and one *ts att *mutation), two of which have been genetically stabilized. The combination of mutations present in these two vaccine candidates should enhance the genetic and phenotypic stability of the viruses, although this will require formal demonstration in a clinical trial using clinical grade virus preparations.

Evaluation of the two vaccine candidates revealed that they are reasonable candidates for further study in clinical trials. Both candidates replicated well in Vero cells, a characteristic that is important for manufacturing purposes. Both viruses also demonstrated a strong *ts *phenotype in vitro (shut-off temperature of ≤38°C) that was similar to that of their *ts *parent virus, but the two viruses differ in their level of temperature sensitivity in vitro. Since the level of temperature sensitivity of respiratory viruses [24], including HPIV1 as demonstrated here, correlates with level of attenuation, it was anticipated that this difference in the *ts *phenotype would be reflected in a difference in the level of attenuation and immunogenicity in vivo, and this indeed was seen. The HPIV1 vaccine candidates were both strongly attenuated in the URT and LRT of AGMs, with rHPIV1-C^R84G/Δ170^HN^T553A^L^Y942A ^replicating to slightly higher levels than the more *ts *rHPIV1-C^R84G/Δ170^HN^T553A^L^Δ1710–11^. Both vaccines were weakly immunogenic and failed to induce a detectable level of serum HAI antibodies in AGMs. A low level of protective efficacy was observed in AGMs immunized with rHPIV1-C^R84G/Δ170^HN^T553A^L^Y942A^, but the rHPIV1-C^R84G/Δ170^HN^T553A^L^Δ1710–11 ^was not protective. This low level of immunogenicity and efficacy was not unexpected since each vaccine was highly restricted in replication and since there is a strong correlation between the level of replication of vaccine virus and its immunogenicity and ability to restrict replication of HPIV1 challenge virus. These results can be interpreted to indicate that the two vaccine candidates are over-attenuated, but we think that this conclusion would be premature. It is likely that these viruses will be more immunogenic, and therefore more efficacious, in humans compared to AGMs since they should replicate more efficiently in humans. The reasons for this are two-fold. First, HPIV1 is a human virus, and it should replicate more efficiently in its natural host in which it causes disease than in AGMs in which it causes only an asymptomatic infection. The actual level of replication of HPIV1 in seronegative humans is unknown, but it replicates efficiently even in adults with pre-existing immunity [25,26]. Second, these vaccine candidates are highly *ts *and should replicate more efficiently in humans, which have a lower body core temperature (36.7°C), than in AGMs (approximately 39°C). Therefore, although these vaccine candidates appear to be over-attenuated in AGMs, it is expected that the viruses should replicate somewhat more efficiently in humans and would be more immunogenic than in AGMs. It also is fortunate that the two vaccine candidates appear to differ somewhat in their level of attenuation, since this provides two chances to achieve an optimal balance between safety and efficacy.

## Conclusion

The rHPIV1-C^R84G/Δ170^HN^T553A^L^Y942A ^and rHPIV1-C^R84G/Δ170^HN^T553A^L^Δ1710–11 ^vaccine candidates are highly attenuated in AGMs. We plan to initiate studies in humans with the less attenuated vaccine candidate, rHPIV1-C^R84G/Δ170^HN^T553A^L^Y942A^. If this virus proves to be insufficiently attenuated in the target population of young seronegative infants (following an initial step-wise progression of safety testing in adults, seropositive children, and seronegative children), we would proceed to evaluate the more attenuated rHPIV1-C^R84G/Δ170^HN^T553A^L^Δ1710–11 ^vaccine candidate. If rHPIV1-C^R84G/Δ170^HN^T553A^L^Y942A ^is over-attenuated, then the L^Y942A ^mutation would be deleted and the rHPIV1-C^R84G/Δ170^HN^T553A ^would be tested in humans. In this way, we will identify a HPIV1 vaccine candidate that exhibits a satisfactory balance between attenuation and immunogenicity for the target population of seronegative infants and young children.

## Methods

### Cells and viruses

LLC-MK2 cells (ATCCCCL7.1) and HEp-2 cells (ATCCCCL23) were maintained in Opti-MEM I (Gibco-Invitrogen, Inc. Grand Island, NY) supplemented with 5% FBS and gentamicin sulfate (50 μg/ml). Vero cells (ATCC CCL-81) were maintained in Opti-PRO SFM (Gibco-Invitrogen, Inc.) in the absence of FBS and supplemented with gentamicin sulfate (50 μg/ml) and L-glutamine (4 mM). BHK-T7 cells, which constitutively express T7 RNA polymerase [27], were kindly provided by Dr. Ulla Buchholz, NIAID, and were maintained in GMEM (Gibco-Invitrogen, Inc.) supplemented with 10% FBS, geneticin (1 mg/ml), MEM amino acids, and L-glutamine (2 mM). Biologically-derived wt HPIV1 Washington/20993/1964, the parent for the recombinant virus system, was isolated previously from a clinical sample in primary African green monkey kidney (AGMK) cells and passaged 2 additional times in primary AGMK cells [25] and once in LLC-MK2 cells [15]. This preparation has a wild type phenotype in AGMs, and will be referred to here as HPIV1 wt. It was previously described as HPIV1_LLC1 _[15]. HPIV1 wt and rHPIV1 mutants were grown in LLC-MK2 cells in the presence of 1.2% Tryple select, a recombinant trypsin (Gibco-Invitrogen, Inc.), as described previously [8].

### Construction of mutant HPIV1 cDNA

P/C, HN and L gene mutations (Table 1) were introduced into the appropriate rHPIV1 subgenomic clones [14] using the Advantage-HF PCR Kit (Clontech Laboratories, Palo Alto, CA) with a modified PCR mutagenesis protocol described elsewhere [28]. The entire PCR amplified subgenomic clone was sequenced using a Perkin-Elmer ABI 3100 sequencer with the Big Dye sequencing kit (Perkin-Elmer Applied Biosystems, Warrington, UK) to confirm that the subclone contained the introduced mutation and to confirm the absence of adventitious mutations introduced during PCR amplification. Full-length antigenomic cDNA clones (FLCs) of HPIV1 containing the desired mutations were assembled using standard molecular cloning techniques [8], and the region containing the introduced mutation in each FLC was sequenced as described above to confirm the presence of the introduced mutation and absence of adventitious changes. Each virus was designed to conform to the rule of six, which is a requirement by HPIV1 and numerous other paramyxoviruses that the nucleotide length of their genome be an even multiple of six for efficient replication [20].

### Recovery of rHPIV1 mutant viruses

Three different recovery methods were used to generate rHPIV1 mutants that differed in the source of the T7 polymerase needed to synthesize RNA from the transfected virus-specific plasmids and, in one case, a different transfection method was used. First, using previously described procedures [8], rHPIV1 virus was recovered from HEp-2 cells that were transfected with plasmids encoding the antigenome and N, P, and L support proteins and infected with an MVA-T7 vaccinia virus recombinant as a source of T7 polymerase. Second, Vero cells were grown to 80% confluency and transfection experiments were performed using the AMAXA Cell Line Nucleofector Kit V, according to manufacturer's directions (AMAXA, Koeln, Germany), as previously described [29]. Briefly, the cells were transfected with 5 μg each of the FLC and the pCL-Neo-BCI-T7 plasmid (expressing T7 polymerase under the control of a eukaryotic promoter) [30], 0.2 μg each of the N and P, and 0.1 μg of the L support plasmids. The transfection mixture was removed after 24 h at 37°C, and cells were washed and overlaid with Opti-PRO with L-glutamine (4 mM) supplemented with 1.2% Tryple select. The cells and supernatant were transferred to LLC-MK2 cells in T25 cm^2 ^flasks (Corning, NY) 7 days following transfection. Third, BHK-T7 cells constitutively expressing T7 polymerase [27] were grown to 90 to 95% confluence in six-well plates. The cells were transfected with 5 μg of the FLC, 0.8 μg each of the N and P, and 0.1 μg of the L support plasmids in a volume of 100 μl of Opti-MEM per well. Transfection was carried out with Lipofectamine 2000 (Invitrogen, Inc., Carlsbad, CA), according to the manufacturer's directions. The transfection mixture was removed after a 24 h incubation period at 37°C, and the cells were washed and maintained in GMEM. On day 2 following transfection, the media was supplemented with 1.2% trypsin, and the recovered virus was harvested on days 2–4. All viruses were amplified by passage on LLC-MK2 cells, and each was cloned by two successive rounds of terminal dilution using LLC-MK2 monolayers in 96-well plates (Costar, Corning Inc., Acton, MA). To confirm that the recovered rHPIV1 mutants contained the appropriate mutations and lacked adventitious mutations, viral RNA (vRNA) was isolated from infected cell supernatants using the Qiaquick vRNA kit (Qiagen Inc., Valencia, CA), reverse transcribed using the SuperScript First-Strand Synthesis System (Invitrogen, Inc., Carlsbad, CA) and amplified using the Advantage cDNA PCR Kit (Clontech Laboratories). Each viral genome was sequenced in its entirety.

### Evaluation of recombinant HPIV1 vaccine candidates in a multiple cycle growth curve

The recombinant HPIV1 mutants were compared to HPIV1 wt on LLC-MK2 and Vero cells at 32°C in a multiple cycle growth curve. Confluent monolayer cultures in 6-well plates were infected in triplicate at a multiplicity of infection (MOI) of 0.01 50%-tissue-culture-infectious-doses (TCID_50_) per cell in media containing trypsin. The residual inoculum was withdrawn 2 h post infection as the day 0 sample and was replaced by medium with trypsin. On days 2, and 4–11 post-infection, the total medium supernatant was removed for virus quantitation and was replaced with fresh medium with trypsin. Supernatants containing virus were frozen at -70°C, and all samples were tested together for virus titer with endpoints identified by hemadsorption.

### Characterization of the temperature sensitivity of the rHPIV1 vaccine candidates

The *ts *phenotype for each mutant rHPIV1 virus was determined by comparing its level of replication to that of HPIV1 wt at 32°C and at 1°C increments from 35°C to 40°C, as described previously [31]. Briefly, each virus was serially diluted 10-fold in 96-well LLC-MK2 monolayer cultures in L-15 media (Gibco-Invitrogen, Inc.) containing trypsin with four replicate wells per plate. Replicate plates were incubated at the temperatures indicated above for seven days, and virus infected wells were detected by hemadsorption with guinea pig erythrocytes. The virus titer at each temperature was determined in three to sixteen separate experiments and is expressed as the mean log_10 _TCID_50_/ml. The mean titer at an elevated temperature was compared to the mean titer at 32°C, and the reduction in mean titer was determined. The shut-off temperature of an rHPIV1 mutant is defined as the lowest temperature at which the reduction in virus titer compared to its titer at 32°C was 100-fold greater than the reduction in HPIV1 wt titer between the same two temperatures. A mutant is defined as having a *ts *phenotype if its shut-off temperature is ≤40°C.

### Evaluation of replication of viruses in AGMs and efficacy against challenge

AGMs in groups of two to four animals at a time were inoculated intranasally (i.n.) and intratracheally (i.t.) with 10^6 ^TCID_50 _of either HPIV1 wt or mutant rHPIV1 in a 1 ml inoculum at each site. NP swab samples were collected daily from days 1 to 10 post-inoculation, and TL fluid samples were collected on days 2, 4, 6, 8 and 10 post-inoculation. The specimens were flash frozen and stored at -80°C and were subsequently assayed in parallel. Virus present in the samples was titered in dilutions on LLC-MK2 cell monolayers in 96-well plates and an undiluted 100 μl aliquot was also tested in 24-well plates. These were incubated at 32°C for 7 days. Virus was detected by hemadsorption, and the mean log_10 _TCID_50_/ml was calculated for each sample day. The limit of detection was 0.5 log_10_TCID_50_/ml. The mean peak titer for each group was calculated using the peak titer for each animal, irrespective of the day of sampling. The mean sum of the virus titers for each group was calculated from the sum, calculated for each animal individually, of the virus titers on each day of sampling, up to day 10. The sum of the lower limit of detectability was 5.0 log_10 _TCID_50_/ml for NP swabs and 2.5 log_10 _TCID_50_/ml for TL samples.

On day 28 post-inoculation, the AGMs were challenged i.n. and i.t. with 10^6 ^TCID_50 _of HPIV1 wt in 1 ml at each site. NP swab and TL samples were collected for virus quantitation on days 2, 4, 6 and 8 post-challenge.

All animal studies were performed under protocol LID22, as approved by the National Institute of Allergy and Infectious Disease (NIAID) Animal Care and Use Committee (ACUC).

### Evaluation of immune responses in AGMs

Serum was collected from each monkey on days 0 and 28 post-immunization and on day 28 post-challenge (day 56 post-immunization). HPIV1 HAI antibody titers were determined at 21°C, as described previously [32], using 0.5% v/v guinea pig erythrocytes and HPIV1 wt as the antigen. The HAI antibody titer was defined as the end-point serum dilution that inhibited hemagglutination and is expressed as the mean reciprocal log_2 _± standard error (SE).

### Statistical Analysis

The Prism 4 (GraphPad Software Inc., San Diego, CA) one-way ANOVA test, (Student-Newman-Keuls multiple comparison test) was used to assess statistically significant differences between data groups (*P *< 0.05). The R software programme [33] was used to perform a Spearman rank test to determine correlation between data sets.

## Competing interests

Patent applications for the vaccine candidates described here have been filed by NIH. In addition, the vaccine candidates are being developed under a Cooperative Research and Development Agreement (CRADA) between NIAID and MedImmune. NIAID investigators work under CRADAs as part of the normal responsibilities of their NIAID, NIH employment. Through the execution of licensing agreements, the NIAID makes the vaccine candidates available to parties interested in their further development and commercialization.

## Authors' contributions

EB recovered viruses, performed in vitro and in vivo studies and drafted the manuscript. AC recovered virus and performed in vitro and in vivo studies. SRS recovered viruses and assisted with in vivo studies. PLC contributed to the study design and drafting of the manuscript. MHS and BRM supervised the study, participated in its design and planning and contributed to drafting of the manuscript. All authors read and approved the final manuscript.
